# Botanical Report

**Published:** 1812-06

**Authors:** 


### BOTANICAL REPORT.

SINCE our last Report three Numbers of the Botanical Magazine have appeared, containing:

DATURA *Metel.* Every species of datura is become a subject of some interest, from the efficacy which the smoking some of them has of late been found to possess, in removing the paroxysms of asthma. In the East Indies, from whence the practice was derived, the roots only were used for this purpose, but the *Stramonium,* the only species common with us, has fibrous roots, on which account the stems have been used, and, very generally, the whole herb, leaves and unripe capsules mixed together. Whether the roots are better for the purpose than the other parts of the plant, Dr. Sims seems to consider as at present undecided. But, if it should be found that a preference is due to this part of the plant, this species appears to be worth cultivating, as its roots are large. Mr. Toulmin, who first witnessed a decided advantage from this remedy in his own person, found the leaves particularly obnoxious to him, but thought the stems of the Stramonium nearly, if not quite, equal to the foreign imported root.

PÆONIA *daurica.* A rare species, the product of the garden of the late Isaac Swainson, esq. the possessor of one of the best botanical gardens in the country.

EDWARDSIA *microphylla.* This elegant shrub has been long known to botanists, under the name of Sophora *microphylla*, but, in the necessary division of this hitherto heterogeneous genus, this has been named by R. A. Salisbury, esq. in honor of Mr. Sydenham Edwards, the able draughtsman for the Botanical Magazine. Sophora *tetraptera* is another species of the same genus.

BRUNSVIGIA *falcata:* generally known as an amaryllis. The species here referred to this genus, besides the one figured, are *multiflora* (Amaryllis orientalis of Jacquin); *marginata; radula* and *striata;* all of which have been figured by Jacquin. According to Mr. Ker, this is really the amaryllis *longifolia* of Linnæus, but not of L'Heritier, the Hortus Kewensis, Jacquin, Willdenow, or the Botanical Magazine, all of which authors mistook the plant figured in the last-mentioned work (No. 661) for it. This plant is from the collection of Lee and Kennedy.

DRIMIA *ciliaris.* Another Cape-bulb, introduced by Mr. William Griffin, of South Lambeth.

TRIGLOCHIN *bulbosum.* From the Cape of Good Hope, from whence it was introduced by George Hibbert, esq. and is now in the collection of Mr. Knight, King's Road, Fulham.

OCIMUM *scutellaroides.* From the collection of John Walker, esq. at Southgate. Dr. Sims has in this article entered into a discussion with Mr. Brown, maintaining against the opinion of the latter, that the corolla of this plant is really resupinate, a question that we shall not undertake to decide.

EURYALE *ferox*, the ANNESLEA *spinosa* of the Botanist's Repository. Although it is true that the latter name was published in the Repository five years after the former one had appeared in the annals, as is here observed by Dr. Sims, yet we believe that this name, in honor of Lord Valentia, was applied by Dr. Roxburgh, and drawings of the plant sent over to this country, even prior to that given by Mr. Salisbury, who probably had seen these drawings at Sir Joseph Banks's, and perhaps ought to have used Dr. Roxburg's name of Anneslea; however, as that of Euryale is adopted in the new edition of the Hortus Kewensis, as well as in the Botanical Magazine, it may be considered hereafter as established. The leaves of this very curious and rare plant float upon the surface of the water, and sometimes exceed three feet in diameter; they are curiously ribbed underneath, and on both sides covered with strong crooked spines. It is cultivated in China, for the sake of its farinaceous seeds, which are said to make a useful article of diet, and the pulp surrounding the seed is applied to medicinal purposes.

GYPSOPHILA *repens.* This species and *prostrata* having been frequently confounded together, we are glad to receive good figures of both in the Magazine, which will hereafter remove all difficulty. They are, however, somewhat too nearly allied.

AMARYLLIS *blanda.* One of the most magnificent species of this splendid genus. Native of the Cape of Good Hope, and has, heretofore, been considered as a variety of A. *belladona;* but Mr. Ker has properly separated it into a distinct species. To this article is added a note on the Amaryllis *jacobæa*, explaining the origin of its trivial name, which Mr. Ker has found from Clusius's Historia Plantarum, was given it by Dr. Simon Tovar, seeing the great resemblance its flower bore to the crimson sword, worn as a badge by the knights of the Spanish order of St. James.

LANTANA *trifolia.* Dr. Sims has remarked the near affinity which this species has with *annüa*, figured in a former number of the Botanical Magazine, and, as Mr. Medicus observed, that this plant produced opposite leaves the first year, and altogether ternate ones on the following; it occurs to us that Lantana *annua*, may probably be nothing more than the same, producing flowers the first year of its growth.

ANTHERICUM *annuum.* A plant of very little beauty; nor does it possess, as far as we have been informed, any other qualifications to render it very worthy of cultivation.

ALOE *albicans.* A very rare species, from Mr. Haworth's collection.

PANCRATIUM *speciosum.* We are informed by Mr. Ker, that this most desirable plant, excelling in beauty and fragrance, has been confounded, in the last edition of the Hortus Kewensis, with P. *caribæum*, of the Botanical Magazine, No. 826, which had been mistaken by Redouté for this species, and published in his Liliacées, under the appellation of *speciosum.*

ANTHERICUM *pugioniforme.* A yellow-flowered species, with round bulb, from the circumference of which issue a row of fusiform roots. Communicated by Mr. Cuff, of Curzon-street.

ALOE *spiralis;* the *imbricata* of Haworth.

BIXA *Orellana.* This is the shrub which produces the coloring drug called Spanish Anotta. This shrub has been very rarely seen in flower in this country. The present drawing was made at the Comtesse de Vandes's collection at Bayswater.

SEMPERVIVUM *soboliferum.* The hen and chicken, as this houseleek is generally called, from its bearing such a numerous offspring, attached to the mother plant by very slender threads, so that they are constantly falling off and taking root, very rarely produces any flowers, unless the young plants are cleared away as fast as they are produced. When the propagation by offsets is in this way prevented, the plant will, for the most part, throw up a flowering stem in the course of the summer.

PIMELEA *rosea*, received from Malcolm and Sweet's nursery, at Kennington Common, is a beautiful little shrub; native of the southern parts of New Holland.

PARNASSIA *caroliniana.* No figure of this American plant appears to have been before published. It is larger than the European species, but less beautiful, on account of the greater simplicity of the nectaries. Communicated by Mr. Gibbs.

We are informed that Mr. Pursh, a German botanist, who has been some years collecting plants in North America, has in the press, a North-American Flora, which will be considerably more extensive than that of Michaux.

